# A dual-branch deep learning framework with Mask-Guided Attention for thyroid nodule classification in ultrasound images

**DOI:** 10.3389/fmed.2026.1694174

**Published:** 2026-03-03

**Authors:** Xueping Liu, Jiajun Zhou, Chuang Xu, Zuojun Fu, Yuwang Zhou, Lulu Jiang, Tianshu Xie, Lei Wu, Yun Fang, Meiyi Yang

**Affiliations:** 1 Quzhou People's Hospital, The Quzhou Affiliated Hospital of Wenzhou Medical University, Quzhou, Zhejiang, China; 2 Yangtze Delta Region Institute (Quzhou), University of Electronic Science and Technology of China, Quzhou, Zhejiang, China; 3 Wenzhou Medical University, Wenzhou, Zhejiang, China; 4 School of Mathematical Sciences, University of Electronic Science and Technology of China, Chengdu, Sichuan, China

**Keywords:** attention mechanism, classification, computer-aided diagnosis, deep learning, lesion segmentation, thyroid nodules, ultrasound imaging, weak supervision

## Abstract

Thyroid nodules are common, and accurate classification into benign or malignant types is essential for effective clinical management. Although high-resolution ultrasound is the primary diagnostic tool, its accuracy is limited by operator dependency. Recent advances in deep learning have shown promise for automated and objective assessment, but many existing methods lack focus on lesion-specific regions, compromising model robustness. To overcome these limitations, we propose a novel dual-branch deep learning framework that combines lesion segmentation and classification. A key feature of this framework is a nodule mask-guided feature enhancement module, which leverages probability masks from the segmentation branch to guide the classification branch toward diagnostically relevant regions while suppressing irrelevant information. Evaluated on ultrasound datasets from three medical centers, our approach demonstrates superior classification accuracy compared to baseline methods, highlighting its potential as a reliable computer-aided diagnosis tool for thyroid nodules.

## 1 Introduction

Thyroid nodules are localized abnormal growths or masses that appear within the thyroid tissues. They are highly prevalent in the general population, with ultrasound examinations detecting thyroid nodules in approximately 19–68% of individuals (1). The prevalence of thyroid nodules shows an age-related increase and is significantly higher in women than in men (2). Among these nodules, approximately 5% are malignant, whereas those with clearly benign features pose an extremely low risk and typically do not require fine-needle aspiration, with follow-up observation being sufficient (3, 4). Failure to detect and treat malignant thyroid nodules on time may lead to serious outcomes, including local invasion, lymph node metastasis, and, in rare cases, distant spread (5). Therefore, accurately distinguishing between benign and malignant thyroid nodules is crucial for avoiding overtreatment and ensuring timely intervention for potentially malignant lesions (6).

High-resolution ultrasound is a commonly used clinical method for evaluating thyroid nodules and is the preferred diagnostic tool due to its non-invasive, cost-effective, and reproducible nature (1, 7). However, the use of ultrasound to distinguish between benign and malignant nodules also presents several challenges in clinical practice. First, the interpretation of ultrasound images depends heavily on the radiologist's experience and expertise, rendering it highly subjective. Thyroid nodules often exhibit heterogeneous appearances and indistinct margins in ultrasound images, which makes accurate and consistent identification of malignant nodules difficult (8). Several studies (9–11) have reported that the diagnostic accuracy of radiologists in predicting the malignancy of thyroid nodules is only approximately 70%–80%. To address these challenges, there is an urgent need to develop computer-aided diagnosis (CAD) systems to assist radiologists in making more reliable assessments.

Early computer-aided diagnosis (CAD) systems in medicine were based on traditional image processing techniques, such as edge detection and threshold segmentation (12). With advances in computational power, machine learning-based CAD systems began to emerge. However, these systems still relied on hand-crafted feature extraction, which introduced a certain degree of subjectivity. These early CAD systems could only serve as auxiliary tools for clinicians rather than providing fully autonomous diagnostic decisions (13). The success of AlexNet (14) in image recognition marked the beginning of widespread application of convolutional neural network (CNN)-based deep learning across various domains. Deep learning enables end-to-end automatic feature learning, overcoming the limitations of traditional CAD systems. As a result, extensive research has focused on developing deep learning-based CAD systems. For example, Gulshan et al. (15) proposed a deep learning model that demonstrated high sensitivity and specificity in detecting diabetic retinopathy and macular edema from retinal fundus photographs. In the BraTS Challenge, Havaei et al. (16) utilized an improved U-Net architecture to achieve automatic segmentation of brain tumors in multimodal MRI scans. These studies highlight the tremendous potential of deep learning in the field of medical image analysis. Wei et al. (17) developed deep learning models for accurate classification of various liver lesions using contrast-enhanced CT images, demonstrating strong generalizability and clinical applicability. Wang et al. (18) proposed a SwinU-based multimodal framework combining MRI-derived features and clinicoradiological information, which achieved strong lymph node risk stratification performance and validated its clinical utility. Wu et al. (19) proposed a patch-based deep learning method for high-precision classification of lung adenocarcinoma growth patterns, generating visualization maps to assist pathologists in diagnosis.

To address the subjectivity and limited accuracy associated with ultrasound-based diagnosis, researchers (20–24) have increasingly introduced deep learning techniques into the recognition and classification of thyroid nodules in recent years. The goal is to enhance diagnostic objectivity and consistency by developing more intelligent, efficient, and robust computer-aided diagnosis (CAD) systems. Although numerous studies have investigated the application of deep learning in ultrasound image analysis of thyroid nodules, several unresolved challenges remain. Most current deep learning-based classification methods use the entire ultrasound image as input, overlooking the varying diagnostic relevance between the lesion and the surrounding background. Given the typically large field of view in ultrasound images, substantial amounts of irrelevant tissue structures or noise may be present. These unrelated features not only interfere with the model's ability to discriminate effectively but may also lead to instability and reduced generalization capacity during feature learning. Therefore, effectively leveraging lesion location information to guide the model's focus toward diagnostically relevant regions–and enhancing the discriminative power of deep features on this basis—is key to improving model performance and reliability. Recent studies have explored integrating lesion localization into classification networks through various strategies to enhance diagnostic performance. Nevertheless, effectively leveraging localization information for classification remains challenging due to uncertain lesion boundaries, heavy annotation requirements, and training instability (25–28).

To address the challenges in thyroid nodule classification, this study proposes a novel dual-branch deep learning architecture guided by lesion location information. The architecture consists of a primary classification branch and an auxiliary segmentation branch. The segmentation branch generates lesion probability masks that directly guide the classification branch to focus on diagnostically relevant regions, enhancing feature representation and improving classification accuracy. This guidance is realized through a Mask-Guided Attention (MGA) module, which uses the segmentation output to selectively emphasize lesion-related features in the intermediate layers of the classification backbone while suppressing irrelevant background information. This explicit spatial attention mechanism helps overcome limitations of traditional CNNs that struggle to capture long-range dependencies and global context, and offers a practical alternative to transformer-based models that require large-scale data and lack supervised localization guidance.

Considering the high cost of pixel-level annotation, we design a weakly supervised segmentation strategy by converting detection bounding boxes into elliptical pseudo-segmentation labels, enabling effective learning of lesion spatial distribution and morphology without expensive manual labeling.

The main contributions of this study are as follows: (1) A dual-branch network combining classification and segmentation with a novel Mask-Guided Attention module to enhance lesion-focused feature learning and improve interpretability. (2) A weakly supervised segmentation framework based on bounding box annotations to reduce annotation burden, validated on a dataset collected from three medical centers, demonstrating the effectiveness and generalizability of the proposed method.

## 2 Material and method

### 2.1 Material

The ultrasound image dataset used in this study was collected from three medical centers: the Quzhou Hospital Affiliated to Wenzhou Medical University (Quzhou People's Hospital), the Quzhou Second People's Hospital, and the Jiangshan People's Hospital. All cases were diagnosed by clinical experts based on postoperative pathology or fine-needle aspiration (FNA) results, ensuring the accuracy and reliability of the labels. Patients were recruited following strict inclusion and exclusion criteria to guarantee data quality and consistency, as detailed below:

(1) Inclusion criteria: Pathological confirmation of papillary thyroid carcinoma (PTC) or benign thyroid nodules through postoperative pathology or FNA; availability of preoperative ultrasound images with at least one view (longitudinal or transverse) of sufficient clarity for analysis; standardized imaging acquisition parameters across centers, including probe frequency and gain settings, to ensure data uniformity.
(2) Exclusion criteria: Incomplete clinical records; poor-quality ultrasound images unsuitable for model training; demographic outliers, specifically patients younger than 14 years or older than 80 years.

For dataset partitioning, cases from the Quzhou Hospital Affiliated to the Wenzhou Medical University and the Quzhou Second People's Hospital were combined to form the training and validation sets. Data from the Jiangshan People's Hospital were reserved as an independent external validation set to assess the generalization capability of the proposed method. Following these criteria, a total of 1,132 patients and 1,943 ultrasound images were included in the experiments. The detailed distribution of training, validation, and external validation sets is summarized in Table 1. All ultrasound images were acquired by experienced medical personnel, and lesion bounding boxes were manually annotated by two senior thyroid ultrasound diagnosticians. To protect patient privacy, all images were anonymized before analysis.

**Table 1:** Statistics of case numbers and image counts for different categories in each dataset.

**Dataset**	**Category**	**Number of cases**	**Number of images**
Training set	Benign nodules	403	659
	Malignant nodules	285	609
Validation set	Benign nodules	193	296
	Malignant nodules	102	225
External validation set	Benign nodules	47	52
	Malignant nodules	102	102

### 2.2 Data preprocessing

The original data format was not standardized, with image sizes varying significantly, ranging from 480 × 640 to 970 × 1,604 pixels. Additionally, the images contained textual descriptions alongside the ultrasound data, necessitating preprocessing. First, adaptive thresholding was applied to binarize the images, turning tissue regions white and irrelevant areas black. Second, morphological operations were used to smooth the images, enhancing the contours of the tissue regions. Third, the largest contour was identified as the tissue area, and a convex hull was applied to the contour to repair any concavities or breaks along the edges. Then, based on the largest contour, a bounding rectangle was generated to crop the tissue region, effectively removing unrelated information. Finally, the cropped tissue region was resized to 224 × 224 pixels to match the input size requirements of the deep neural network.

### 2.3 Method

In this study, we aim to improve the classification of thyroid nodules in ultrasound images by directly leveraging segmentation outputs as spatial priors to enhance the network's focus on diagnostically relevant regions. In clinical practice, the nodule and its surrounding context provide the most critical information for diagnosis. For instance, various thyroid imaging reporting and data systems (TI-RADS) (7, 29) evaluate nodule risk based on key ultrasound features, such as composition, echogenicity, shape, margin, and calcification, all of which depend on accurately identifying the nodule's location and boundaries. However, conventional classification models often process the entire image, where redundant background information can dilute diagnostic cues (30). To address this, we incorporate a segmentation branch that provides spatial guidance to the classification network, thereby enhancing deep features in key regions and improving both discriminative performance and generalization.

#### 2.3.1 Overall architecture

**Figure 1:**
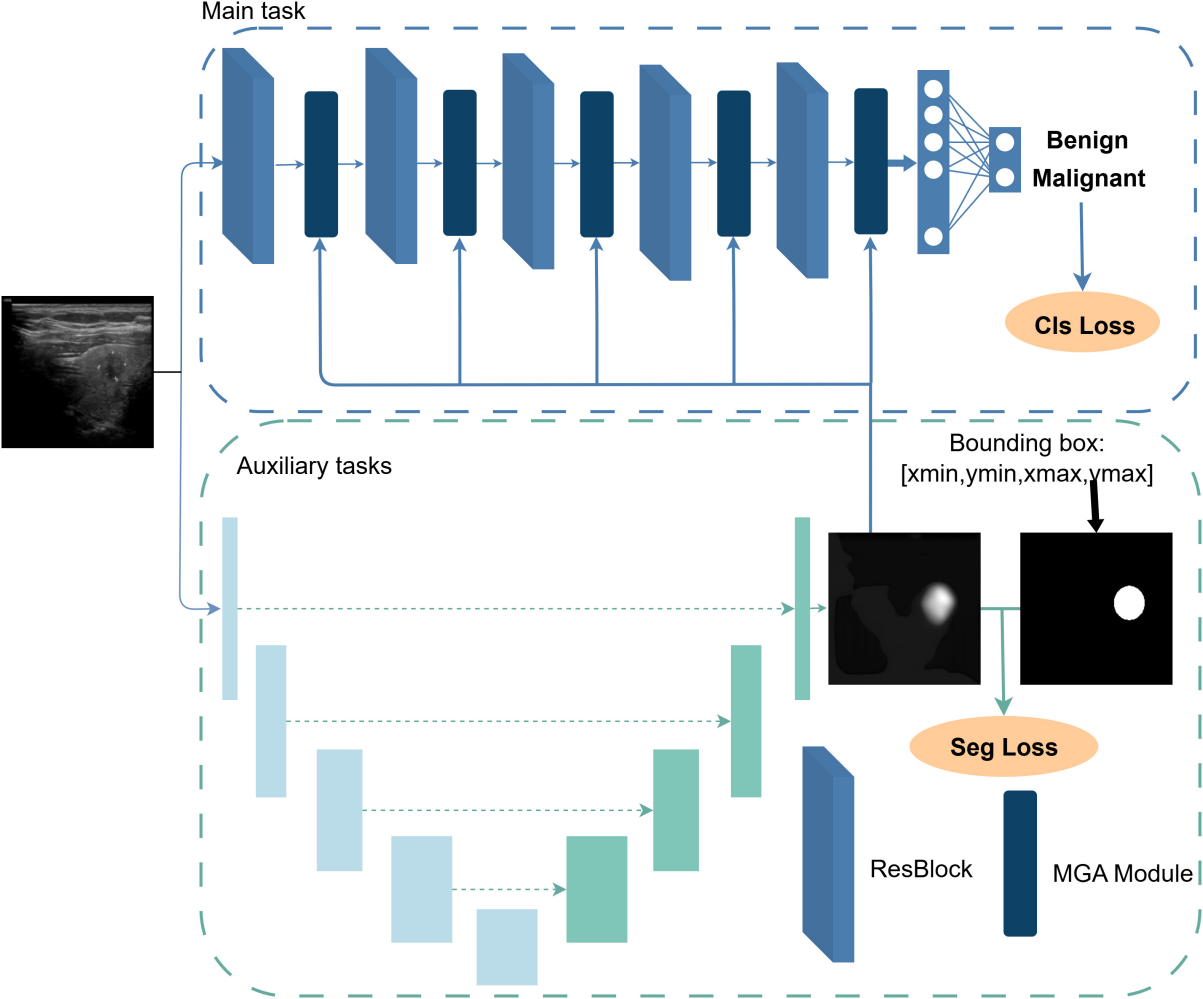
Overall architecture of the proposed MGA-Net, comprising a ResNet34-based classification branch and a ResNet18-based segmentation branch. The segmentation branch guides the classification branch via the MGA module to focus on diagnostically relevant nodule regions.

The proposed network architecture consists of a classification network with ResNet34 (31) as the backbone and a UNet-based (32) segmentation network with ResNet18 as the backbone, as illustrated in Figure 1. The classification network serves as the primary task, aiming to determine the benign or malignant nature of thyroid nodules from patient ultrasound images. The segmentation network acts as an auxiliary task, predicting the spatial distribution of nodules to enhance features that are strongly associated with nodule malignancy classification. Specifically, the segmentation network generates a probability map for each pixel indicating the likelihood of belonging to the key region (nodule area). This mask is then fed into a mask-guided feature enhancement module to strengthen the intermediate features of the classification network (ablation experiments were conducted to investigate which intermediate layers benefit most from such enhancement). Notably, this segmentation-guided design not only encourages the model to focus more on learning lesion-specific features during training but also increases the relative weight of these features in the classification process during validation. This dual-branch architecture explicitly decouples localization from recognition, delegating the localization task to the auxiliary branch so that the classification branch can focus solely on extracting critical texture and shape features of the lesion, thereby improving the accuracy of thyroid nodule classification while also providing a degree of interpretability.

Moreover, the dual-branch network is trained in a staged joint manner. In the first stage, the segmentation model is pre-trained to stabilize the subsequent training of the classification network. In the second stage, both branches are trained jointly. The classification loss backpropagates through the feature enhancement module to the segmentation branch, encouraging the predicted masks to better align with classification needs, thereby achieving collaborative optimization of segmentation and classification.

#### 2.3.2 Mask-Guided Attention module

Traditional convolutional neural networks (CNNs) have been widely used for image classification tasks, including thyroid ultrasound analysis. However, CNNs often struggle to capture long-range dependencies and global context, limiting their ability to focus on diagnostically relevant regions (33). Recently, transformer-based models, such as Vision Transformer (ViT) (34), have shown promise by modeling global relationships through self-attention mechanisms. While ViT can theoretically highlight key areas by considering interactions between image patches, it requires large amounts of training data and lacks explicit supervised guidance to focus on lesion regions. These limitations hinder the practical application of such models in medical imaging, where annotated data are limited and precise localization is crucial. In addition to transformer-based approaches, several attention mechanisms have been introduced to enhance CNN representations, among which the Squeeze-and-Excitation (SE) block (35) and the Convolutional Block Attention Module (CBAM) (36) are the most representative. SE focuses on modeling channel-wise dependencies by reweighting feature channels using global pooling statistics, while CBAM further sequentially incorporates spatial attention based on feature-derived responses. However, these methods rely on implicit attention inferred from feature statistics and lack explicit lesion-level supervision, which limits their ability to directly guide the network toward diagnostically relevant regions in medical imaging tasks. Therefore, designing attention mechanisms distinct from ViT that explicitly guide CNN-based models to focus on diagnostically important regions is essential for improving both classification accuracy and interpretability in thyroid nodule diagnosis.

To address this, we propose a nodule mask-guided feature enhancement module. This module uses the probability mask generated by the segmentation branch to guide the intermediate feature layers of the classification backbone to focus on regions with high nodule probability while suppressing irrelevant information. During training, the module enables the model to concentrate more on lesion areas, learning more effective and generalizable classification features, thereby reducing the risk of shortcut learning. In the testing phase, the module also helps the model leverage more critical deep features, resulting in improved recognition performance.

**Figure 2:**
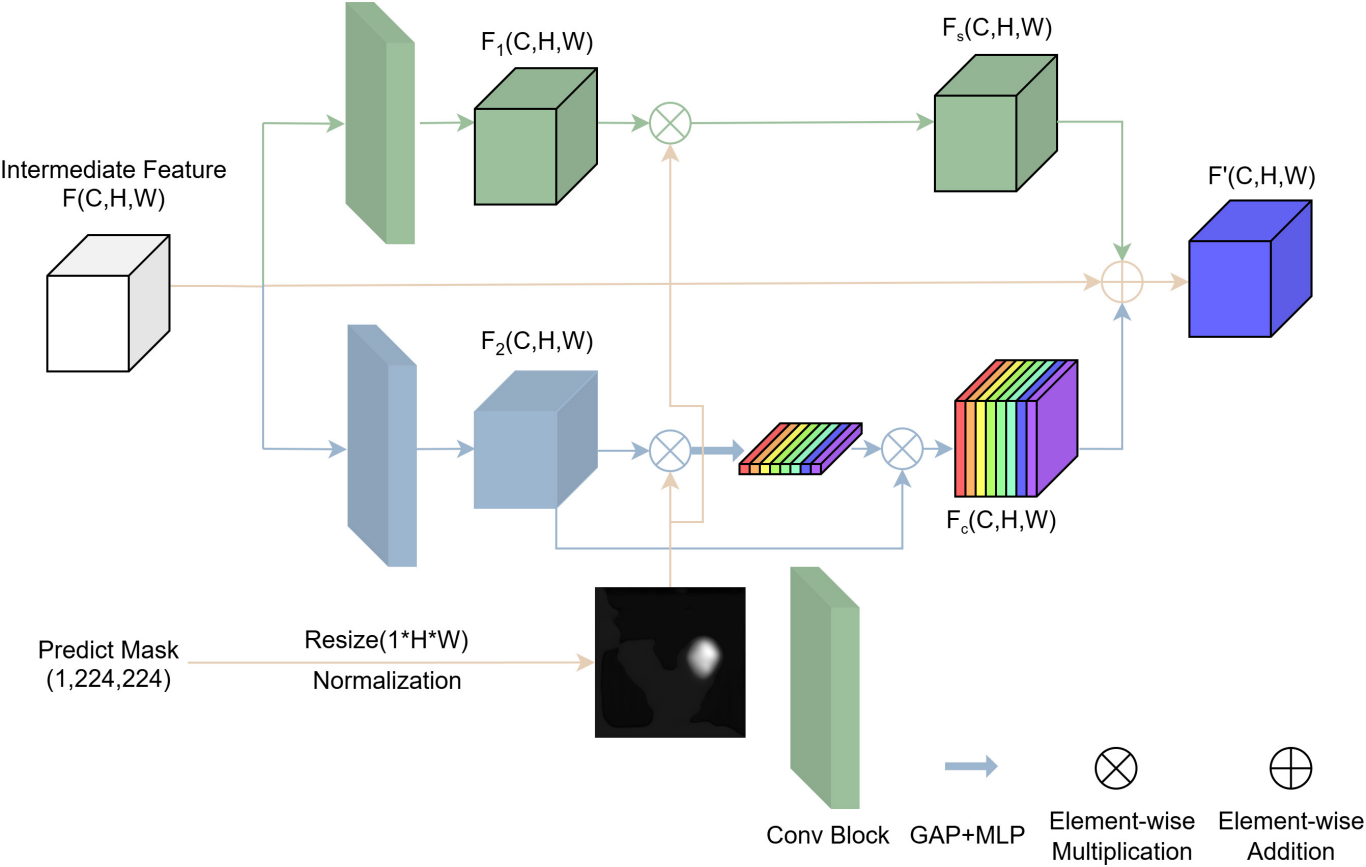
Structure diagram of the Mask-Guided Attention (MGA) module.

As shown in Figure 2, the proposed feature enhancement module consists of two submodules: a spatial attention enhancement module and a channel attention enhancement module. These submodules specifically enhance local features that are strongly correlated with nodule characteristics from spatial and channel dimensions, respectively. The two submodules operate in parallel, and their enhanced features are added element-wise and combined with the original intermediate features via residual connections.

Specifically, the input to this module is an intermediate feature map *F*(*C, H, W*) and a mask *mask*(1 × 224 × 224) generated by the segmentation branch. First, the mask is resized to match the dimensions of the intermediate feature map using bilinear interpolation, resulting in *mask*_*resized*_(1 × *H*×*W*). This resized mask is then normalized to the range [0,1] through min-max normalization, yielding the probability mask *mask*_*norm*_.


$$mask_{resized}=Resize\left(mask,H,W\right),\;mask_{resized}\in {\mathbb{R}}^{1\times H\times W}$$

$$mask_{norm}\left(i,j\right)=\frac{mask_{resized}\left(i,j\right)-\min\left(mask_{resized}\right)}{\max\left(mask_{resized}\right)-\min\left(mask_{resized}\right)+\epsilon }$$

Here, ϵ is a small constant (e.g., 10^−6^) used to avoid division by zero.

The goal of the spatial attention branch is to highlight key nodule regions in the spatial dimension of the feature map while suppressing irrelevant background regions, thereby guiding the feature learning of the network to focus more effectively. Based on clinical priors, the mask is considered an important decision-making region and is directly used to enhance the corresponding positions in the intermediate features.


$$F_{s}=F⊙mask_{norm}$$

The purpose of the channel attention branch is to identify relatively more important feature channels using the mask and enhance these channels, enabling the model to focus more on critical features. Specifically, this branch first extracts key features *feature*_*enhance*_(*C, H, W*) using the mask and then applies global average pooling followed by a multi-layer perceptron (MLP) to obtain the attention coefficients for each channel.


$$F_{c}=F⊙MLP\left(GAP\left(F⊙mask_{norm}\right)\right)$$

The two attention submodules are combined in parallel to form the mask-guided feature enhancement module. The final output of this module is obtained by fusing the features enhanced by both attention branches, as follows:


$$F^{\prime }=F_{s}+F_{c}+F$$

#### 2.3.3 Loss

The loss functions used in this study consist of two components: the classification loss for the primary task and the segmentation loss for the auxiliary task. The classification loss not only optimizes the backbone of the classification branch for feature extraction but also updates the entire segmentation branch through the mask-guided feature enhancement module. The overall loss function used in this study is defined as follows, where the classification loss is formulated as a cross-entropy loss:


$$\begin{array}{l} L_{total}=L_{cls}+L_{seg} \end{array}$$ (1)

$$\begin{array}{l} L_{cls}=-\overset{C}{\underset{c=1}{\sum }}y_{c}\log\left({ŷ}_{c}\right) \end{array}$$ (2)

In designing the auxiliary task, we carefully weighed the applicability and performance of using a detection branch vs. a segmentation branch and ultimately opted for a segmentation branch constructed from detection labels, rather than directly employing an object detection branch. There are several key reasons for this choice. First, the essence of an object detection task is to locate the approximate position of a target, focusing on its presence and coarse boundaries, typically annotated in the form of bounding boxes. In medical imaging, this approach can roughly cover the lesion area but pays insufficient attention to the precise boundary information of the lesion. Detection branches are relatively insensitive to fine-grained variations in texture and shape, making them less capable of capturing complex structures—particularly in scenarios where lesion boundaries are blurred or irregular—leading to significant performance limitations. In contrast, segmentation tasks require the model to make pixel-level predictions across the entire image, thereby exhibiting greater sensitivity to texture variations at the boundary regions. This enables the segmentation branch to drive the model to learn richer and more detailed local features, which is especially beneficial in improving the identification of key lesion regions when boundaries between lesions and surrounding tissues are blurred in medical images.

However, pixel-level mask annotation in medical imaging is costly, and high-quality manual segmentation labels are not always available in clinical practice. To address this issue, we leverage existing detection labels to generate regular elliptical pseudo-labels within the bounding boxes as weakly supervised masks for training the segmentation branch. This strategy avoids the high cost of manual segmentation while preserving the segmentation branch's ability to model boundary features, thereby enabling fine-grained modeling of key regions under weak supervision. The conversion of a detection box [*x*_*min*_, *y*_*min*_, *x*_*max*_, *y*_*max*_] into a segmentation label is formulated as follows:


$$\begin{array}{l} \left(x_{c},y_{c}\right)=\left(\frac{x_{\min}+x_{\max}}{2},\frac{y_{\min}+y_{\max}}{2}\right) \end{array}$$ (3)

$$\begin{array}{l} \left(a,b\right)=\left(\frac{x_{\max}-x_{\min}}{2},\frac{y_{\max}-y_{\min}}{2}\right) \end{array}$$ (4)

$$\begin{array}{l} mask\left(i,j\right)=\{\begin{array}{ll} 1, & if\left(\frac{{\left(j-x_{c}\right)}^{2}}{a^{2}}+\frac{{\left(i-y_{c}\right)}^{2}}{b^{2}}\right)\leq 1\\ 0, & otherwise \end{array} \end{array}$$ (5)

Therefore, we argue that a segmentation branch based on detection-derived pseudo-labels can, without increasing the annotation burden, more effectively leverage the boundary-awareness capability of segmentation tasks, thereby improving the overall task performance. The segmentation loss (37) is defined as:


$$\begin{array}{l} L_{seg}=\alpha \cdot L_{BCE}+\left(1-\alpha \right)\cdot L_{Dice} \end{array}$$ (6)

$$\begin{array}{l} L_{BCE}=-\frac{1}{N}\overset{H}{\underset{i=1}{\sum }}\overset{W}{\underset{j=1}{\sum }}[mask\left(i,j\right)\log\left(ŷ\left(i,j\right)\right)+(1\\ \;-mask\left(i,j)\right)\log(1-ŷ\left(i,j\right))] \end{array}$$ (7)

$$\begin{array}{l} L_{Dice}=1-\frac{2\sum_{i=1}^{H}\sum_{j=1}^{W}mask\left(i,j\right)\cdot ŷ\left(i,j\right)+\epsilon }{\sum_{i=1}^{H}\sum_{j=1}^{W}mask\left(i,j\right)+\sum_{i=1}^{H}\sum_{j=1}^{W}ŷ\left(i,j\right)+\epsilon } \end{array}$$ (8)

### 2.4 Evaluation metrics

To comprehensively evaluate the diagnostic performance of the proposed model in the classification of benign and malignant thyroid nodules, three key metrics were selected: accuracy (ACC), F1 score, and the area under the receiver operating characteristic curve (AUC). For each metric, the 95% confidence interval (95% CI) was calculated to reflect the statistical robustness of the model's performance. Among these, the F1 score was regarded as the primary evaluation metric, as it jointly considers precision and recall for the positive class, making it particularly informative for tasks with imbalanced class distributions or those emphasizing the detection of malignant cases. A higher F1 score indicates superior overall performance of the model in identifying malignant nodules.


$$\begin{array}{l} F1score=\frac{2TP}{2TP+FP+FN} \end{array}$$ (9)

Accuracy reflects the overall prediction correctness, i.e., the proportion of correctly classified samples to the total number of samples. The AUC, calculated from the ROC curve, measures the model's overall discriminative ability across various decision thresholds, with values closer to 1 indicating superior capability in distinguishing benign from malignant nodules. All metrics are reported as the mean values of five independent experiments, along with their corresponding 95% confidence intervals (95% CI), to assess the reliability and statistical significance of the results, particularly in scenarios with limited sample sizes or potential performance fluctuations.

### 2.5 Experimental setup

To evaluate the proposed method, all experiments were implemented using PyTorch and conducted on an NVIDIA GeForce RTX 3090 GPU. Model parameters were optimized using the Adam optimizer (38) with an initial learning rate of 0.0001. Random horizontal flipping was applied as a data augmentation strategy to improve the model's robustness to variations in nodule orientation. The training process consisted of two stages: the segmentation branch was first pre-trained independently for 60 epochs to enhance training stability, followed by joint training of both segmentation and classification branches for an additional 140 epochs with a batch size of 32, resulting in a total of 200 training epochs. In the joint training stage, the total loss was defined as a weighted combination of the classification loss and the segmentation loss, with the balancing coefficient α set to 0.5. All comparative methods were trained under identical experimental settings to ensure a fair and reliable evaluation.

## 3 Results

### 3.1 Comparative experiment result

In this study, we compared our method with several popular classification architectures, including three convolution-based models, Inception (39), VGG (40), and ResNext (41), as well as two Vision Transformer (ViT)-based models, ViT (34) and SwinTransformer (42). The results of the three main performance metrics on the validation set are shown in Table 2, where our method outperforms all five comparison methods across all metrics. Among the three convolution-based baselines, their performances are relatively close, with ResNext achieving the best results of 0.8998, 0.8848, and 0.9582 for accuracy, F1 score, and AUC, respectively. Our method, also convolution-based, shows clear performance advantages over ResNext, with improvements of 3.2%, 3.48%, and 1.67% in accuracy, F1 score, and AUC, respectively. For the two transformer-based baselines, the performance difference is more pronounced: ViT demonstrates relatively poor classification performance, while SwinTransformer achieves comparatively higher scores. Our method significantly outperforms both transformer-based models. Compared to ViT, we achieve improvements of 8.33%, 10.07%, and 4.60% in accuracy, F1 score, and AUC, respectively. Compared to SwinTransformer, our method improves these metrics by 4.63%, 4.75%, and 3.22%, respectively.

**Table 2:** Mean values and 95% confidence intervals of accuracy, AUC, and F1 score for each method.

**Method**	**Accuracy (95% CI)**	**AUC (95% CI)**	**F1 score (95% CI)**
Inception	0.8987 (0.8960, 0.9013)	0.9568 (0.9511, 0.9624)	0.8829 (0.8788, 0.8871)
VGG	0.8821 (0.8782, 0.8861)	0.9451 (0.9353, 0.9549)	0.8649 (0.8592, 0.8705)
ResNeXt	0.8998 (0.8939, 0.9057)	0.9582 (0.9488, 0.9676)	0.8848 (0.8794, 0.8902)
ViT	0.8572 (0.8471, 0.8673)	0.9314 (0.9236, 0.9391)	0.8318 (0.8216, 0.8421)
Swin	0.8875 (0.8839, 0.8911)	0.9438 (0.9369, 0.9506)	0.8741 (0.8706, 0.8775)
Proposed	0.9286 (0.9247, 0.9325)	0.9742 (0.9694, 0.9790)	0.9156 (0.9103, 0.9210)

**Figure 3:**
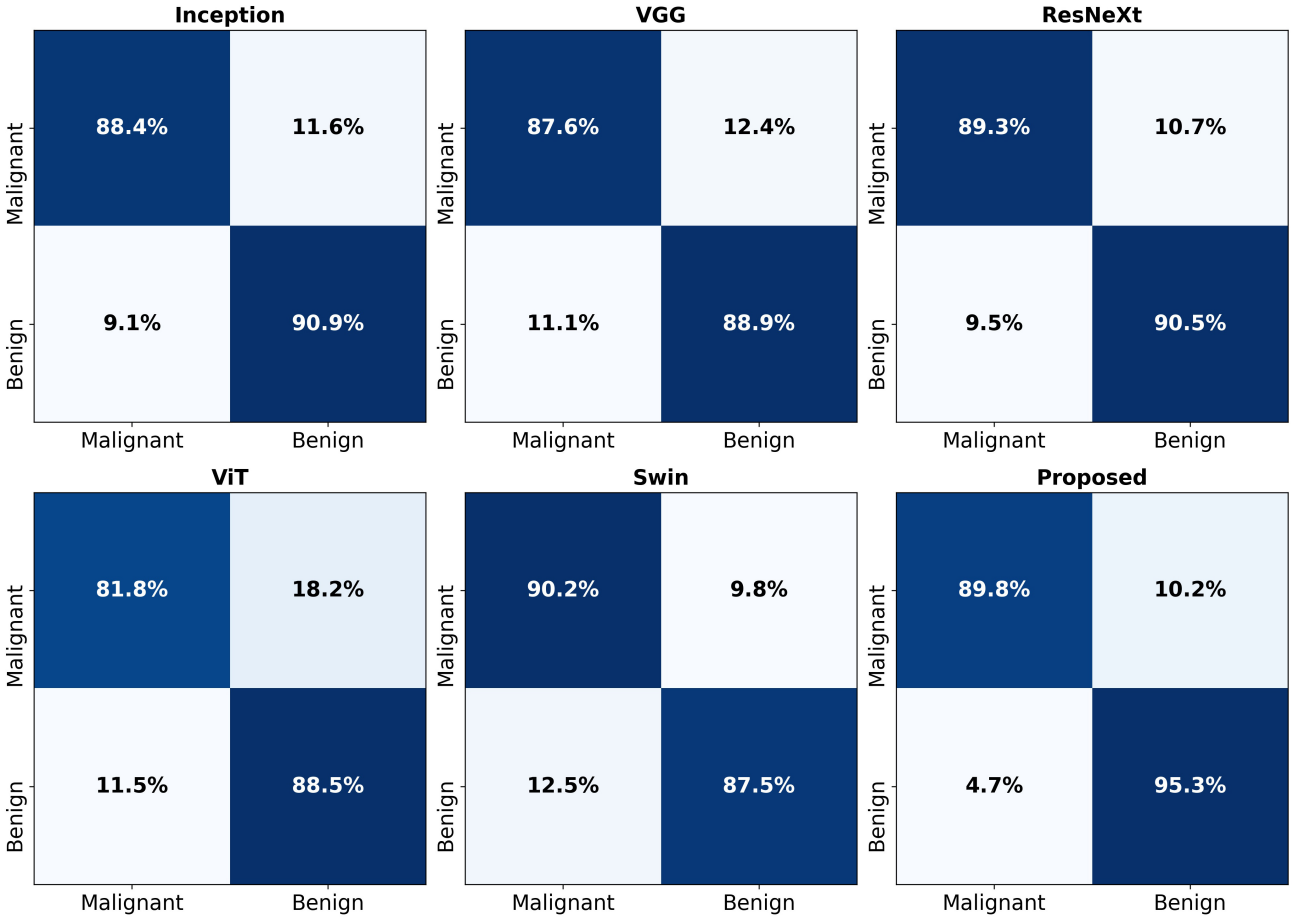
Confusion matrices expressed as percentages for different models. Each subplot shows the prediction performance of the model in classifying benign and malignant thyroid nodules. The color intensity represents the percentage of correct predictions, with white numbers indicating exact values.

Figure 3 presents the confusion matrices of the proposed method (Proposed) and five comparative models for the benign and malignant thyroid nodule classification task. Compared to other methods, the Proposed model demonstrates higher accuracy in identifying both benign and malignant nodules, with a notable advantage in significantly reducing false positives (i.e., benign nodules misclassified as malignant). This helps to lower the risk of unnecessary clinical interventions. These results further validate the superior practical applicability of our method.

### 3.2 Ablation experiment results

To further validate the effectiveness of the proposed components and design choices, we conducted a series of ablation experiments aimed at evaluating the independent contributions of key modules and verifying the rationality of their integration strategy.

#### 3.2.1 Effectiveness of the feature enhancement module

This section aims to verify the effectiveness of the core module proposed in this study–the mask-guided feature enhancement module. This module consists of a spatial attention submodule and a channel attention submodule, which are integrated in parallel. To assess the roles of the two submodules and the rationality of their parallel architecture, we designed three ablation experiments and compared them with the final scheme. Specifically, these include: using only the spatial attention submodule, using only the channel attention submodule, connecting the two submodules in series, and the complete scheme containing both parallel submodules.

As shown in Table 3, the baseline model without any enhancement module (Base) achieved an F1 score of 0.8642, while adding only channel attention or only spatial attention both brought significant performance improvements. Among them, the variant with only spatial attention (F1 = 0.9039) outperformed the variant with only channel attention (F1 = 0.8741), indicating that spatial information plays a more critical role in our task.

**Table 3:** Classification performance of different attention structures on the validation set.

**Setting**	**Accuracy (95% CI)**	**AUC (95% CI)**	**F1 score (95% CI)**
Base	0.8829 (0.8805, 0.8853)	0.9478 (0.9426, 0.9531)	0.8642 (0.8605, 0.8680)
Channel-only	0.8929 (0.8883, 0.8975)	0.9542 (0.9466, 0.9618)	0.8741 (0.8690, 0.8793)
Spatial-only	0.9182 (0.9118, 0.9247)	0.9703 (0.9625, 0.9782)	0.9039 (0.8964, 0.9114)
Serial fusion	0.9044 (0.8985, 0.9103)	0.9657 (0.9601, 0.9713)	0.8866 (0.8762, 0.8970)
Proposed	0.9286 (0.9247, 0.9325)	0.9742 (0.9694, 0.9790)	0.9156 (0.9103, 0.9210)

Base, without enhancement module; Channel-only, channel attention only; Spatial-only, spatial attention only; Serial fusion, serial connection; Proposed, parallel connection. Values in parentheses represent 95% confidence intervals.

Moreover, we compared the serial and parallel fusion strategies of the two attention submodules. The serial structure showed improvement over single submodules, achieving an F1 score of 0.8866. However, our proposed parallel design performed best across all metrics, including accuracy (0.9286), AUC (0.9742), and F1 score (0.9156), significantly surpassing the serial fusion. These results confirm the necessity of integrating spatial and channel attention as well as the effectiveness of the parallel fusion design, which allows the two submodules to complement each other without interfering with their respective attention mechanisms.

To investigate which feature enhancement strategies are more effective in improving classification accuracy, we designed two ablation experiments. The first strategy applies feature enhancement after every convolutional block to achieve finer-grained enhancement. The second strategy performs enhancement only once after the last block of each layer, i.e., enhancing features at each scale only once. Under this setting, we further conducted layer-wise ablation experiments to analyze the impact of enhancement modules at different layers on model performance, thereby verifying the necessity of enhancement at each layer.

From Table 4, it can be seen that all enhancement settings bring performance improvements compared to the Base (no enhancement). The EB setting improves accuracy, AUC, and F1 by approximately 0.9%, 0.4%, and 1.1%, respectively. The Proposed setting further improves these metrics to 0.9286, 0.9742, and 0.9156, indicating that the enhancement modules significantly boost the model's classification performance, particularly by better extracting discriminative features. Comparing the two enhancement strategies, EB (enhancement after every block) and Proposed (enhancement once at the end of each layer), the Proposed approach outperforms EB across all metrics. Although fewer enhancement positions are used, the Proposed method achieves more notable improvements. This suggests that applying enhancement at the end of each feature scale more effectively integrates semantic information, realizing more efficient feature enhancement than dense enhancement after every block. For the Proposed setting, we performed a layer-wise ablation by removing the enhancement module at a specific scale (i.e., -L0 to -L4) to analyze the contribution of each layer. The results show that removing enhancement from any layer leads to performance degradation, especially when Layer 2 and Layer 4 are removed, indicating that enhancement at mid-to-high level layers contributes more to the final classification performance. This validates the necessity of enhancement modules at all layers and highlights the importance of multi-scale feature enhancement.

**Table 4:** Classification performance of different enhancement settings on the validation set.

**Setting**	**Accuracy (95% CI)**	**AUC (95% CI)**	**F1 (95% CI)**
Base	0.8829 (0.8805, 0.8853)	0.9478 (0.9426, 0.9531)	0.8642 (0.8605, 0.8680)
EB	0.8914 (0.8864, 0.8963)	0.9519 (0.9477, 0.9562)	0.8751 (0.8678, 0.8824)
-L0	0.9225 (0.9212, 0.9238)	0.9707 (0.9656, 0.9759)	0.9087 (0.9069, 0.9105)
-L1	0.9190 (0.9170, 0.9210)	0.9693 (0.9634, 0.9752)	0.9047 (0.9025, 0.9070)
-L2	0.9228 (0.9208, 0.9248)	0.9693 (0.9667, 0.9720)	0.9088 (0.9066, 0.9109)
-L3	0.9194 (0.9160, 0.9228)	0.9697 (0.9650, 0.9743)	0.9053 (0.8999, 0.9106)
-L4	0.9152 (0.9132, 0.9172)	0.9693 (0.9667, 0.9719)	0.8996 (0.8965, 0.9026)
Proposed	0.9286 (0.9247, 0.9325)	0.9742 (0.9694, 0.9790)	0.9156 (0.9103, 0.9210)

Base, no enhancement; EB, enhancement after every block; -Lk, removal of enhancement at layer k (scale), others retained; Proposed, enhancement modules included at layers 0-4.

### 3.3 External validation

This section mainly demonstrates the effectiveness of our method compared with a series of baseline and competing methods on an external validation dataset, aiming to verify the generalizability of our approach.

As shown in Table 5, our proposed method demonstrates significantly superior performance compared to the baseline methods on the external validation set, indicating good generalization ability. Specifically, the proposed method achieves accuracy, AUC, and F1 acore of 0.8130, 0.8835, and 0.8507, respectively, all of which are notably higher than those of all comparison models. Among them, traditional CNN architectures (such as Inception, VGG, and ResNeXt) show some competitiveness on certain metrics but overall lag significantly behind. Transformer-based architectures, including ViT and Swin Transformer, perform poorly on this task, especially ViT, whose AUC is only 0.5896, possibly due to its limited ability to model small sample sizes or structural details. In contrast, our method effectively improves the modeling of complex boundaries and fine-grained features by introducing multi-scale feature fusion and mask-guided feature enhancement strategies, resulting in more robust classification performance on external data.

**Table 5:** Classification performance of various methods on the external validation set.

**Method**	**Accuracy (95% CI)**	**AUC (95% CI)**	**F1 score (95% CI)**
Inception	0.6844 (0.6539, 0.7149)	0.7676 (0.7291, 0.8060)	0.7287 (0.6896, 0.7679)
VGG	0.6987 (0.6785, 0.7189)	0.7717 (0.7594, 0.7840)	0.7627 (0.7315, 0.7939)
ResNeXt	0.7324 (0.7067, 0.7401)	0.7816 (0.7428, 0.8204)	0.7908 (0.7768, 0.8047)
ViT	0.6857 (0.6813, 0.6901)	0.7093 (0.6769, 0.7418)	0.7624 (0.7430, 0.7819)
Swin	0.7130 (0.7025, 0.7235)	0.7542 (0.7118, 0.7965)	0.7644 (0.7486, 0.7801)
Proposed	0.8130 (0.8010, 0.8249)	0.8835 (0.8474, 0.9197)	0.8507 (0.8375, 0.8640)

### 3.4 Visualization

**Figure 4:**
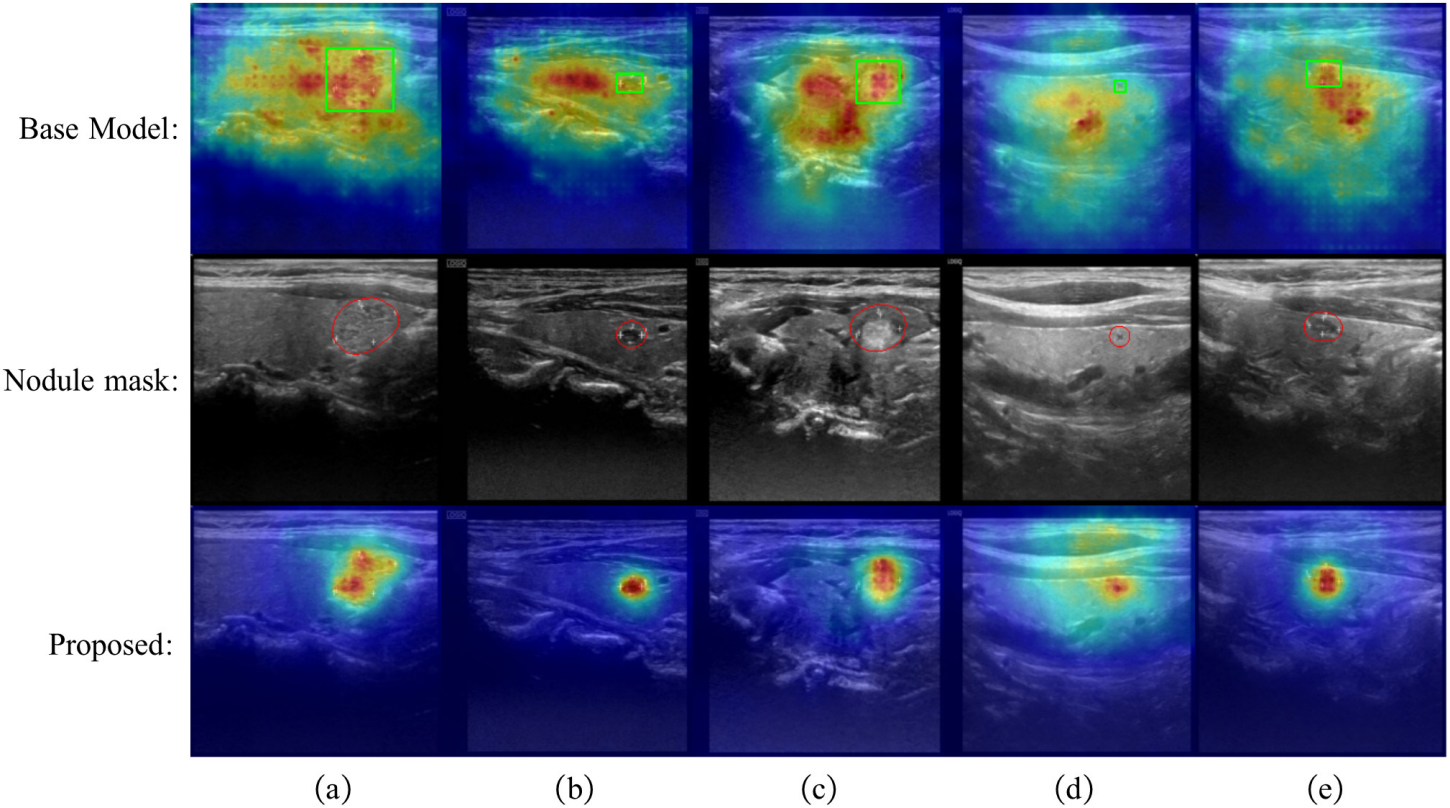
Visualization results. The green boxes indicating clinician-annotated lesions are shown in the first row.

To verify that our segmentation branch can effectively help the classification branch focus on key regions, we present in Figure 4 the activation maps of the baseline model, the masks generated by the segmentation branch in our method, and the final classification activation maps (CAM) (43, 44). From the visualization results, it is evident that the baseline model has a limited ability to focus on lesion areas. For some samples (e.g., b and d), the lesion locations are barely identified, while in samples a, c, and e, although the activation regions cover parts of the lesions, a considerable amount of attention is still dispersed to irrelevant areas far from the lesions. This indicates that without explicit guidance, the model lacks sufficient feature focusing, which may lead to attention being directedto unrelated regions and thus affect the final classification performance.

In contrast, the segmentation branch in the proposed method is able to accurately localize lesion regions. Although some boundary errors exist, the overall coverage is good (contextual information near the nodules may also contain important features). Based on this mask information, the classification branch is able to focus on the lesion and its surrounding critical regions during inference, effectively suppressing interference from irrelevant features. The final CAM maps exhibit stronger discriminability and regional consistency, demonstrating that the model can make more accurate classification decisions based on the semantic features of the lesion areas. Overall, the results validate that the segmentation branch, aided by the mask-guided enhancement module, effectively enhances the feature representation of the classification branch and improves classification performance.

## 4 Discussion

In recent years, the widespread application of high-resolution ultrasound has significantly increased the detection rate of thyroid nodules. However, it has also led to a large number of low-risk or benign nodules being identified, which poses challenges for clinical classification and risk assessment (1, 45, 46). Given that ultrasound interpretation relies heavily on experience and has limited accuracy, this study proposes an automated method that integrates segmentation and classification based on the clinical prior that “the nodule and its surrounding region contain key information for distinguishing benign from malignant” (7, 29). The method explicitly enhances the multi-scale features of the classification branch with nodule masks generated by the segmentation branch, enabling the model to focus more on lesion areas during training and validation, thereby improving the accuracy of benign-malignant discrimination (47, 48).

Previous studies have attempted to leverage the positional information of thyroid nodules to improve classification performance. For example, Kang et al. (25) proposed a consistency learning framework combining intra-task and inter-task consistency, establishing constraints between the prediction layers of segmentation and classification tasks to enhance thyroid nodule classification accuracy; Zhao et al. (26) designed a TAD module to decouple tissue and anatomical features, enabling the model to simultaneously learn both feature types, effectively enhancing ultrasound image classification through segmentation; Tang et al. (27) introduced a shape feature extraction module based on contour detection and fused it with texture feature streams to enhance the classification network's utilization of nodular structural information.

However, these studies have two main limitations: First, the understanding of segmentation tasks is mostly limited to “learning segmentation features to assist classification,” while segmentation supervision essentially guides the model to learn shape patterns and regional consistency of lesions. How these segmentation features specifically influence the classification task remains unclear. In contrast, directly using segmentation results to guide the classification network may be a more intuitive and effective approach. Second, these methods heavily rely on networks fully learning segmentation features, requiring comprehensive pixel-level segmentation annotations, which are difficult to obtain in practice and may limit the applicability of these methods.

To address these shortcomings, this study proposes a multi-task learning framework that explicitly uses segmentation results to guide classification. By introducing a segmentation branch in the network and using approximate segmentation masks generated from detection boxes as supervision, the model learns salient lesion locations, morphology, and regional consistency during training. Through a mask-guided multi-scale feature enhancement module, the outputs of the segmentation branch are deeply integrated with the classification branch. Notably, our method does not depend on costly pixel-level segmentation labels but only requires relatively simple and easily obtainable lesion bounding boxes (four coordinates), effectively reducing annotation difficulty and cost. This design allows the classification branch to focus more on lesion areas and their surrounding critical contextual information during feature extraction, effectively suppressing background interference and improving classification accuracy and robustness.

Extensive comparative and ablation experiments fully validate the effectiveness and superiority of the proposed mask-guided multi-scale feature enhancement module in thyroid nodule benign-malignant classification. Compared with multiple classical convolutional networks (Inception, VGG, and ResNeXt) and mainstream Transformer architectures (ViT and Swin Transformer), our method significantly improves key metrics, such as accuracy, AUC, and F1 score, demonstrating good generalization on external validation datasets, reflecting the model's robustness and practical value. Ablation studies further reveal the complementary roles of spatial and channel attention submodules in feature enhancement and verify the advantages of the parallel fusion structure. These findings indicate that a well-designed feature enhancement strategy can effectively increase the model's attention to critical lesion regions, thereby strengthening the representation of discriminative features. Moreover, layer-wise ablation analysis shows that multi-scale feature enhancement is crucial for improving classification performance, especially the contribution of mid- and high-level features, highlighting the synergistic effect of semantic information across different layers. The inclusion of a segmentation branch in the multi-task architecture provides additional spatial localization supervision during training, helping the classification branch focus on lesion regions and reducing reliance on irrelevant background. Visualization results intuitively demonstrate the effectiveness of this mechanism, further supporting the conclusion that the mask-guided feature enhancement module improves the model's discriminative power.

Despite the promising performance, there remain areas for improvement. First, the absence of precise segmentation masks prevents us from investigating the impact of accurate segmentation labels vs. detection box-generated pseudo masks on feature enhancement. Second, our current method mainly addresses the binary classification of thyroid nodules (benign vs. malignant) and has not yet been extended to finer-grained subtype recognition or multi-class diagnosis.

In summary, the proposed mask-guided multi-scale feature enhancement strategy not only significantly improves the classification performance of thyroid nodule malignancy but also provides a valuable reference for effectively utilizing spatial localization information in medical image classification tasks, with considerable clinical application potential and scalability.

## 5 Conclusion

This study proposes a mask-guided multi-scale feature enhancement strategy that deeply integrates approximate segmentation masks generated from detection boxes with the classification branch, achieving significant improvements in thyroid nodule benign-malignant classification. By introducing a parallel fusion module combining spatial and channel attention, the model focuses more on lesions and their critical contextual information during feature extraction, effectively suppressing background interference. Compared to various mainstream convolutional networks and transformer architectures, the proposed method demonstrates superior performance on accuracy, AUC, and F1 score metrics and achieves good generalization on external validation datasets.

## Data Availability

The datasets presented in this article are not readily available because the datasets generated and/or analyzed during the current study are not publicly available due to patient privacy and ethical restrictions. Requests for access to anonymized data cannot be accommodated to ensure compliance with institutional and regulatory guidelines. Requests to access the datasets should be directed to Meiyi Yang, meiyiyang@csj.uestc.edu.cn.
